# Contrast Media Volume Control and Acute Kidney Injury in Acute Coronary Syndrome: Rationale and Design of the REMEDIAL IV Trial

**DOI:** 10.1016/j.jscai.2023.100980

**Published:** 2023-04-28

**Authors:** Carlo Briguori, Enrica Mariano, Alessandro D’Agostino, Mario Scarpelli, Amelia Focaccio, Salvatore Evola, Giovanni Esposito, Giuseppe Massimo Sangiorgi

**Affiliations:** a Interventional Cardiology Unit, Mediterranea Cardiocentro, Naples, Italy; b Dipartimento di Biomedicina e Prevenzione, Università Tor Vergata, Rome, Italy; c Division of Cardiology, Paolo Giaccone University Hospital, Palermo, Italy; d Division of Cardiology, Department of Advanced Biomedical Science, “Federico II” University of Naples, Naples, Italy

**Keywords:** acute coronary syndrome, acute kidney injury, contrast media

## Abstract

**Background:**

Although the pathogenesis of acute kidney injury (AKI) in patients with acute coronary syndrome (ACS) undergoing invasive treatment is multifactorial, the role of iodinated contrast media (CM) has been well established. The DyeVert system (Osprey Medical) is designed to reduce the CM volume during invasive coronary procedures while maintaining fluoroscopic image quality.

**Objective:**

The aim of the Renal Insufficiency Following Contrast Media Administration Trial IV (REMEDIAL IV) is to test whether the use of the DyeVert system is effective in reducing contrast-associated acute kidney injury (CA-AKI) rate in patients with ACS undergoing urgent invasive procedures.

**Trial Design:**

Patients with ACS treated by urgent invasive approach will be enrolled. Participants will be randomly assigned into one of the following groups: (1) DyeVert group and (2) control group. In participants enrolled in the DyeVert group, CM injection will be handled by the DyeVert system. On the contrary, in the control group, CM injection will be performed by a conventional manual or automatic injection syringe. In all cases, iobitridol (a low-osmolar, nonionic CM) will be administered. Participants will receive intravenous 0.9% sodium chloride as soon as moved to the catheterization laboratory. The primary end points are CM volume administration and CA-AKI rate (ie, an increase in serum creatinine concentration of ≥0.3 mg/dL within 48 hours after CM exposure). A sample size of at least 522 randomized participants (261 in each group) is needed to demonstrate an 8.5% difference in the CA-AKI rate between the groups (that is, from 19% in the control group to 10.5% in the DyeVert group), with a 2-sided 95% confidence interval and 80% power (*P* < .05).

## Highlights

• Acute kidney injury occurs in acute coronary syndrome patients treated invasively.
• Contrast media minimization is crucial for limiting acute kidney injury.
• The DyeVert system reduces contrast media volume during coronary procedures.
• The DyeVert system use may reduce acute kidney injury rate in patients with acute coronary syndrome.

## Central Illustration

**Figure:**
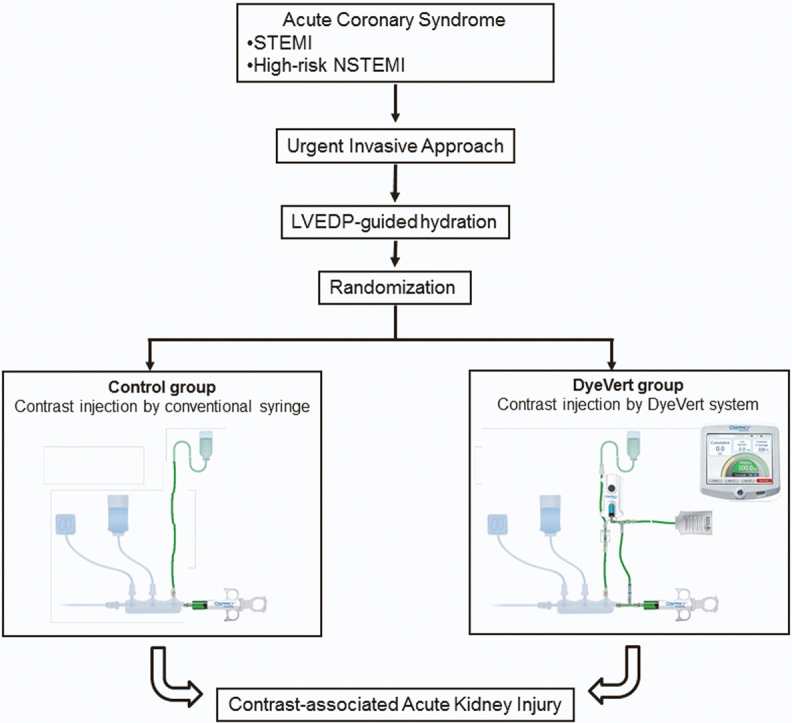

## Introduction

Acute kidney injury (AKI) is a common complication in patients with acute coronary syndrome (ACS), being treated by invasive approach.^1^^,^^2^ This complication has been associated with higher early and late adverse events.^3^ Although the pathogenesis of AKI in patients with ACS is multifactorial,^4^ the role of iodinated contrast media (CM) has been well established.^5^ Volume expansion represents the cornerstone in contrast-associated acute kidney injury (CA-AKI) prevention.^6^ However, all recommended volume expansion regimens have limited applicability in patients with ST-elevation myocardial infarction (STEMI) and high-risk non–ST-elevation myocardial infarction (NSTEMI) transferred to percutaneous coronary intervention (PCI)-capable centers for emergency invasive treatment. Therefore, in this scenario, it is of outmost importance to limit the CM volume in the attempt to prevent CA-AKI. The DyeVert system (Osprey Medical) is a novel device designed to reduce the CM volume during coronary procedures, while maintaining fluoroscopic image quality.^7^

The aim of the Renal Insufficiency Following Contrast Media Administration Trial IV (REMEDIAL IV) is to test whether the use of the DyeVert system is effective in reducing AKI rate in patients with ACS undergoing urgent invasive diagnostic and interventional coronary procedures.

## Methods

### Recruitment, enrollment, and allocation

This multicenter, randomized, investigator-driven, clinical trial will assess the role of the DyeVert device in limiting AKI rate in patients with ACS. All patients with ACS scheduled for urgent/immediate invasive approach will be screened for inclusion/exclusion criteria (Tables 1 and 2). Diagnosis of ACS (both STEMI and high-risk NSTEMI) will be established in accordance with guidelines, including a typical chest pain history, diagnostic electrocardiographic changes, and serial increase in cardiac biomarker concentrations.^8^, ^9^, ^10^ All patients with inclusion/exclusion criteria satisfied and who will agree to sign the informed consent form will be enrolled into the study. The REMEDIAL IV will be conducted at 4 Italian interventional cardiology centers (Supplemental Methods and Supplemental Table S1). Radial access will be recommended. The study is registered with www.ClinicalTrials.gov (NCT04714736). The number of participants screened, treated, and analyzed will be reported according to the CONSORT guidelines.

**Table 1:** Inclusion criteria

Condition	Definition
Age ≥18 y	–
Acute coronary syndrome	ST-elevation myocardial infarction^a^ High-risk non–ST-elevation myocardial infarction^b^: a) Refractory angina b) Signs or symptoms of heart failure or new or worsening mitral regurgitation c) Hemodynamic instability d) Recurrent angina or ischemia at rest or with low-level activities despite intensive medical therapy e) Sustained VT or VF f) Recurrent dynamic ST-T wave changes, particularly with intermittent ST-elevation
Urgent or immediate (within 2 h) invasive approach	Coronary procedure with iodinated contrast media administration

VT, ventricular tachycardia; VF, ventricular fibrillation.

a According to the Fourth Universal Definition of Myocardial Infarction.^8^

b According to the current guidelines.^9^^,^^10^

**Table 2:** Exclusion criteria

Condition	Definition
Women who are pregnant	–
Recent contrast media exposure	Contrast media exposure within 48 h
End-stage CKD on chronic dialysis	Both hemodialysis and peritoneal dialysis
Multiple myeloma	–
Patients referred from a spoke center for an invasive treatment but not hospitalized in the institutions conducting the study	–
Current enrollment in any other study when enrollment in the REMEDIAL IV would involve deviation from either protocol	–

CKD, chronic kidney disease; REMEDIAL IV, Renal Insufficiency Following Contrast Media Administration Trial IV.

### Protocol

Participants included into the study will be randomly assigned into 2 groups (Central Illustration): (1) DyeVert group and (2) control group.

**Central Illustration:**
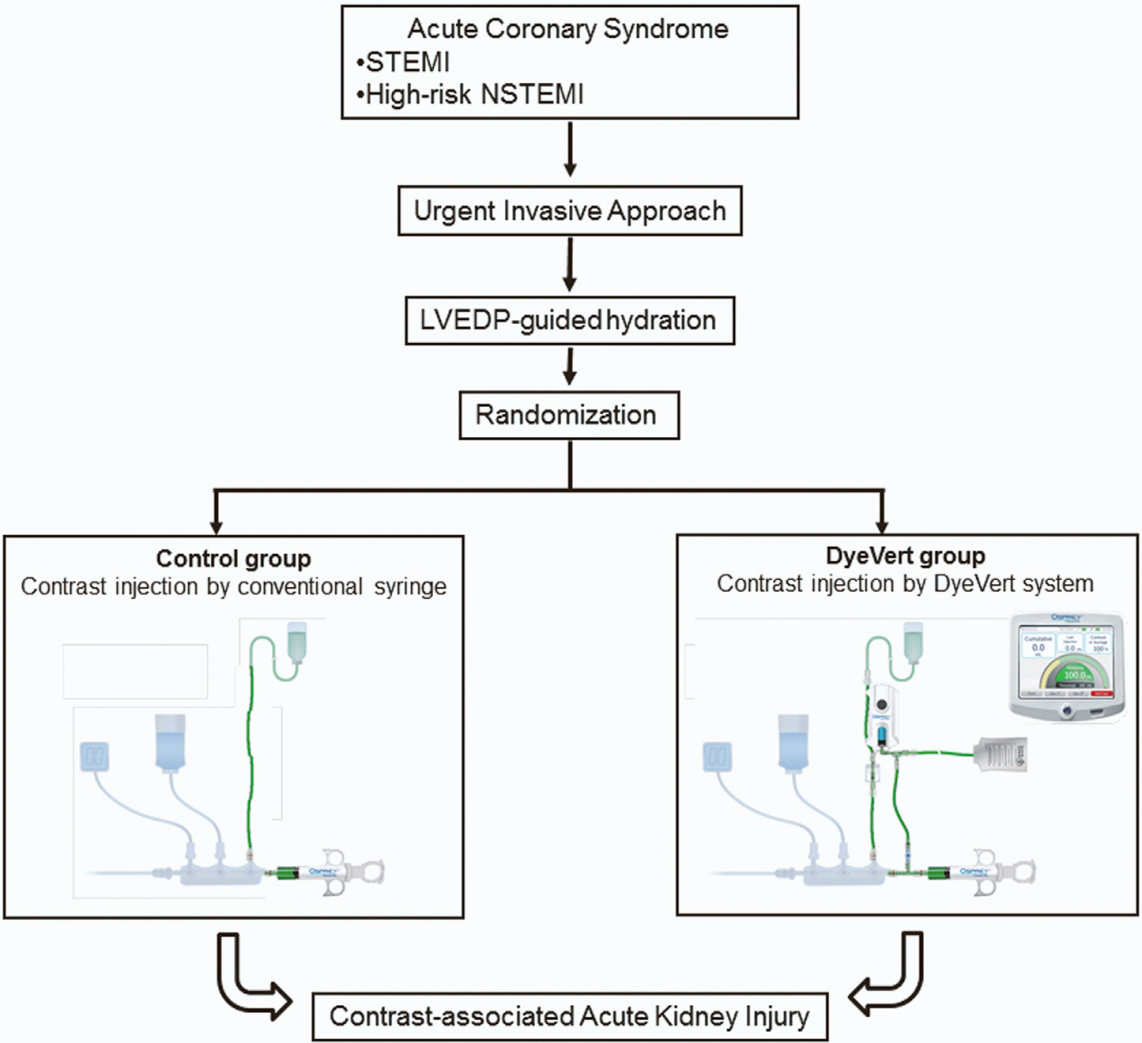
**Schematic representation of the study design.** STEMI, ST-elevation myocardial infarction; NSTEMI, non–ST-elevation myocardial infarction; LVEDP, left ventricular end-diastolic pressure.

#### The DyeVert group

In participants enrolled in this group, CM injection will be handled by the DyeVert system. The DyeVert system (Osprey Medical) is a device designed to reduce the CM volume during coronary procedures, while maintaining fluoroscopic image quality.^7^ The DyeVert Plus EZ Contrast Reduction Systems is compatible with manual contrast injection, whereas the DyeVert Power XT Contrast Reduction System is compatible with automated contrast injection (ACIST; ACIST Medical Systems). (Supplemental Methods). It allows for the modulation of the CM volume during an injection. During an injection, the DyeVert system diverts a portion of the injected CM through a secondary fluid pathway controlled by a pressure-compensating diversion valve. This allows a decrease in overinjection of CM and less aortic reflux. The diversion valve provides variable resistance in the secondary fluid path to ensure a flow rate to the patient, which results in an adequate image quality. The valve is constructed in a way that the diversion pathway resistance automatically increases with higher injection pressures and decreases with lower injection pressures, proportionally decreasing or increasing CM delivered to patients, respectively. The diverted CM is temporarily stored in the reservoir and is returned to the injection syringe when the physician aspirates CM for the next injection. The associated Contrast Monitoring System (Osprey Medical) displays CM volume injected (in milliliters), split in attempted, delivered, and saved (the last reported as both absolute value and percentage vs the total). The DyeVert system can be used in conjunction with 4F to 6F diagnostic catheters and 5F to 7F guide catheters.

#### The control group

CM injection in this group will be performed by a conventional manual injection syringe or automatic injection device (ACIST Medical Systems).

### Iodinated contrast media

Iobitridol (Xenetix 350; Guerbet; 350 mg iodine/mL), a nonionic, low-osmolality (915 mOsm per kilogram of water) CM, will be used in all participants. Strategies for limiting the CM volume are as follows^11^: (1) in the control group, angiograms will be performed with an injection of CM using a 10-cm^3^ syringe; this provides strict control of CM delivery by limiting the volume of contrast that can be administered in a single injection; (2) catheters with side holes will be strictly avoided during PCI; (3) when exchanging catheters, unused CM is withdrawn from the catheter lumen (eg, by back-bleeding through an opened “Y”-connector or by aspirating residual CM from the catheter using a syringe); (4) “tests” with “puffs” of CM are discouraged; and (5) left ventriculography will not be permitted. The administration of a CM volume of >3× glomerular filtration rate (GFR) is suggestive of increased risk of CA-AKI.^12^ A new CM source bottle will be used in the control group when using manual syringe injection to be sure about the starting CM volume level. After the procedure, the end volume on the CM source bottle will be marked to indicate the CM volume ending point, and the amount of remaining CM within the syringe will be documented. Then, the total volume of CM used from the bottle will be measured using a graduated cylinder.

### Volume expansion regimen

Normal saline (0.9% sodium chloride) will be used in all instances. The volume expansion rate will be 3 mL/kg/h and will start as soon as the patient will arrive into the catheterization laboratory. Volume expansion will continue during the procedure and for at least 6 hours postprocedure. However, in case of hemodynamic instability (Supplemental Methods and Supplemental Table S2), volume expansion will be performed at the rate of ≤1.5 mL/Kg/h or, if deemed clinically contraindicated, will not be started at all. Intraprocedural volume expansion will be guided by left ventricular end-diastolic pressure (LVEDP), according to the Prevention of Contrast Renal Injury with Different Hydration Strategies (POSEIDON) trial^13^ (Supplemental Table S3). LVEDP measurement will be made in all patients by placing a 5F or 6F pigtail catheter in the midcavity of the left ventricle at the beginning of the procedure and before CM injection. LVEDP will be measured at the beginning of the isometric ventricular contraction at the “Z” point. A total volume expansion of >960 mL is considered the optimal cutoff volume to prevent CA-AKI.^2^

### Biomarkers of renal function and injury

Serum creatinine (sCr), cystatin C, blood urea nitrogen, sodium, and potassium concentrations will be measured at the baseline (ie, as soon as the participant will arrive in the emergency room or in the catheterization laboratory room before CM injection) and every day during the hospital stay until discharge; additional measurements will be performed in all cases of deterioration of baseline renal function. Estimated GFR will be calculated by applying the Chronic Kidney Disease Epidemiology Collaboration (CKD-EPI) equation.^14^ Chronic kidney disease is defined as a GFR of <60 mL/min/1.73 m^2^. The risk scores for predicting CA-AKI will be estimated according to the Mehran score^15^ and Gurm score^16^ (Supplemental Methods and Supplemental Tables S4 and S5).

### Study end points

The primary end point of the trial are CM volume and the rate of AKI, defined as an increase in a sCr concentration of ≥0.3 mg/dL within 48 hours after CM administration or the need for dialysis.^17^ Secondary end points will include the following: (1) an increase in the sCr concentration of ≥0.5 and/or ≥25% mg/dL within 72 hours after CM exposure; (2) the severity of AKI assessed according to the KDIGO criteria^17^: stage 1/risk, a sCr concentration increase of ≥0.3 mg/dL or ≥1.5-1.9 times from the baseline level; stage 2/injury, a sCr concentration increase of ≥2.0-2.9 times from the baseline; and stage 3/failure, a sCr concentration increase of ≥3.0 times from the baseline or the need for dialysis; (3) changes in the serum cystatin C concentration at 24 and 48 hours after CM exposure; (4) the rate of acute renal failure requiring dialysis (defined as a decrease in renal function necessitating acute hemodialysis, ultrafiltration, or peritoneal dialysis within the first 5 days postintervention); (5) the length of in-hospital stay, calculated as the sum of the number of days since admission until discharge from the hospital; (6) the rate of in-hospital and 1-month, 6-month, and 12-month major adverse events, such as death, major bleeding, renal failure requiring dialysis, and sustained kidney injury. Major bleeding will be defined according to the BARC criteria.^18^ Sustained kidney injury is defined as a persistent ≥25% GFR reduction, compared with baseline and the last available value during the follow-up.^19^

### Data collection and monitoring

Demographic characteristics, a medical history, and current medication of all participants will be recorded at the baseline. Total hydration volume administered according to the prophylaxis and during 24 and 48 hours after the procedure and the total urine volume will be recorded. The preprocedure sCr level is considered as the baseline value before the initiation of any prophylaxis. Participant records will include the presence of clinical parameters of hemodynamic instability. These include the following: (1) left ventricular ejection fraction (LVEF), (2) LVEDP, (3) critical state (defined as a cardiogenic shock requiring treatment with positive inotropes, need for intra-aortic balloon counterpulsation treatment, or mechanical ventilation), (4) heart failure episodes treated conservatively, (5) clinically significant tachyarrhythmias (ventricular fibrillation, sustained ventricular tachycardia, and atrial fibrillation), and bradyarrhythmias requiring pacemaker, and (6) major bleeding. Bleeding will be defined according to the BARC criteria.^18^ Cardiogenic shock will be defined according to the Society for Cardiovascular Angiography and Interventions (SCAI) classification (Supplemental Table S6).^20^ End point data and adverse events will be collected during the in-hospital stay and at 1-month, 6-month, and 12-month major adverse events. All adverse events will be recorded in the case report form, and the data coordinating center will be informed by facsimile within 72 hours of any events. Serious events and any other safety issues will be reviewed by an independent data monitoring and safety committee. All events will be adjudicated by a clinical events committee (CEC), blinded to the treatment assignment. At least 2 members of the CEC will review clinical data and relevant documentation and will determine whether end points have occurred according to the study definitions. In case of disagreement between the reviewers, a third member of the CEC will adjudicate, and the data will be considered by the entire committee if 2 of the 3 reviewers do not agree.

### Statistical analysis

Efficacy analyses will be based on an intention-to-treat strategy defined as all subjects randomly assigned, regardless of the treatment actually received. Treatment allocation to the 2 groups will be determined by randomization in a 1:1 ratio. To ensure that almost equal number of participants will receive one of the 2 treatments, randomization blocks of 4 will be used. An independent statistician will generate the randomization list with permuted blocks, and the block size will be not disclosed to the investigators enrolling the participants (Random Allocation Software 1.0). Categorical data will be summarized as mean and standard deviation. Dichotomous outcomes will be compared by χ^2^ test, using exact procedures. According to the published data, the expected AKI rate in the control group is 19%.^1^, ^2^, ^3^^,^^21^^,^^22^ The sample size has been estimated to test the hypothesis that the reduction in the CM volume obtained by the DyeVert system would translate into an absolute difference of 8.5% and a 45% relative risk reduction in AKI rate between the groups.^12^^,^^23^ Therefore, a sample size of 261 participants in each group (a total of ≤522 randomized participants) is needed to demonstrate an absolute reduction in AKI rate from 19.0% in the control group to 10.5% in the DyeVert group, with a 2-sided 95% confidence interval and 80% power (*P* < .05); based on the large sample normal approximation extended 0.07 from the observed difference in proportions. The test statistic used will be the 2-sided Fisher exact test. The significance level of the test will be .050. A prespecified subgroup analysis includes the following: ACS type (STEMI vs NSTEMI), GFR (<60 vs ≥60 mL/min/1.73 m^2^), LVEF (<40% vs ≥40%), diabetes mellitus, baseline risk score, hydration volume (<960 vs ≥960 mL), manual versus automatic injection, multivessel stent placement, cardiogenic shock, sex, and age (younger than 75 years vs 75 years or older).

## Discussion

Patients with ACS are at high risk of AKI: in this subset of patients, indeed, the reported AKI rate ranges from 15 to 30%.^1^, ^2^, ^3^^,^^24^ The pathogenesis of AKI in the setting of ACS is multifactorial. Age, unstable hemodynamic conditions, comorbidities (ie, diabetes mellitus and anemia), preexisting chronic renal disease, dehydration, and administration of nephrotoxic drugs may concur in the development of AKI.^4^ However, the role of CM has been well established.^5^

Volume expansion represents the cornerstone in CA-AKI prevention.^6^ Currently, there is no consensus on how volume expansion should be performed, particularly in patients with ACS. The most recommended regimen is normal saline infusion at 1 mL/kg/h (0.5 mL/kg/h if LVEF ≤35% or NYHA >2) from 12 hours or before to 24 hours after CM exposure.^25^ However, this regimen is not suitable in urgent/emergent settings. Maioli et al^2^ suggested that early rapid volume expansion (3 mL/kg/h starting in the emergency department), followed by infusion of 1 mL/kg/h for 12 hours, allowing to a mean value of almost 1200 mL, is effective in preventing CA-AKI in STEMI patients. However, this regimen is contraindicated in patients with ACS and unstable hemodynamic conditions: in this clinical scenario, a forced volume expansion regimen may increase the risk of pulmonary edema. In the “a Maastricht contrast-induced nephropathy guideline (AMACING)” trial (which showed that, in 660 patients deemed to be at risk of CA-AKI, no prophylaxis was noninferior to intravenous hydration for the prevention of AKI and was also cost-saving), 4% of patients in the hydration group experienced complications that led to hydration being stopped prematurely. This rate is quite high and unexpected. In studies that enrolled patients at higher risk of AKI than those included in the AMACING trial, the reported rate of pulmonary edema was substantially lower (1%-1.5%).^12^^,^^13^^,^^22^ These findings reinforce the concept of tailored volume expansion protocols—a single protocol should not be applied to all patients. Several tailored volume expansion regimens have been proposed, such as those guided by LVEDP,^13^ urine flow rate,^24^ central venous pressure,^23^ and bioimpedence.^26^ All these protocols have been shown to be superior to the conventional recommended volume expansion regimen for prevention of CA-AKI and are associated with a reduced risk of pulmonary edema. In this trial, we will adopt the LVEDP-guided protocol because this approach is simple and easy to implement in our target population (ie, patients with ACS undergoing an invasive approach).

CM volume administered is an independent predictor of CA-AKI.^5^^,^^27^ Owing to the nonlinear relationship between CM volume and AKI, some “safe” thresholds have been proposed (Supplemental Table S7). However, others have reported a linear relationship between CM volume and AKI rate.^21^^,^^28^ It has been reported that a 30% reduction in CM volume could translate into a 12.8% reduction in AKI.^21^ SCAI expert consensus statement on best practices in the cardiac catheterization laboratory emphasizes quality indicators related to CM volume reduction and monitoring.^11^ To date, the use of manual injections with a manifold remains the preferred technique in most catheterization laboratories.^29^ Although CM flow rate and maximum injection pressure can be preset in automatic systems, the manual syringe provides constant pressure feedback to the operator that might favor better modulation of the injection. The “a clinical trial for contrast media volume reduction and incidence of CIN” (AVERT) tested the efficacy of the DyeVert system to reduce the CM volume used during coronary angiographic procedures without impairing image quality and to prevent AKI.^30^ The AVERT trial demonstrated that CM volume is significantly lower in patients receiving DyeVert in comparison with control subjects (36.9 ± 10.9 mL vs 62.5 ± 12.7 mL; *P* < .001). This resulted in a 41% decrease in the volume of CM used. The observed reduction in CM volume used was most evident in patients who underwent PCI. The volume of CM spared with the use of AVERT was directly related to the complexity of the PCI procedure, with 31% and 46% reductions in CM volume used when 2 and 3 lesions were treated, respectively.

Other methods for CM volume minimization have been tested. However, biplane angiography has not been proven to significantly reduce CM volume injected.^31^ Moreover, in the minimizing contrast utilization with IVUS guidance in coronary angioplasty trial (MOZART), intravascular ultrasound–guided PCI reduced the CM volume used compared with angiography-guided PCI.^32^ However, intravascular ultrasound is not widely used and can increase procedure costs and duration.

Preliminary data suggest that the DyeVert system is effective in reducing CM volume and AKI rate in patients with ACS undergoing invasive treatment.^12^ The REMEDIAL IV will clarify whether the DyeVert system is an effective strategy to reduce both CM volume and AKI rate in patients with ACS undergoing urgent invasive procedures.
