# Supplementary material for: Contrast Media Volume Control and Acute Kidney Injury in Acute Coronary Syndrome: Rationale and Design of the REMEDIAL IV Trial
Source: J Soc Cardiovasc Angiogr Interv. 2023 Apr 28;2(4):100980. doi: 10.1016/j.jscai.2023.100980 (PMC11307588; doi:10.1016/j.jscai.2023.100980)
Supplement: Supplemental Methods [file mmc1.docx]

**SUPPLEMENTARY METHODS**

**Table S1. REMEDIAL IV centers**

| **Centre** | **Investigators** |
| --- | --- |
| Interventional Cardiology Unit, Mediterranea Cardiocentro, Naples, Italy | Dr. Carlo Briguori |
| Department of Advanced Biomedical Science, Division of Cardiology, “Federico II” University, Naples, Italy | Prof. Giovanni Esposito |
| Department of Biomedicine and Prevention, Tor Vergata University, Rome, Italy | Dr. Giuseppe Massimo Sangiorgi  Dr.ssa Enrica Mariano |
| Division of Cardiology, Paolo Giaccone University Hospital, Palermo, Italy | Dr. Salvatore Evola  Dr. Alessandro D’Agostino |

**Table S2. Criteria for hemodynamic instability**

| **Condition** | **Definition** |
| --- | --- |
| Pulmonary congestion | Mild–moderate (Killip class 2) to overt pulmonary edema (Killip class 3) |
| Low cardiac output state | Tachicardia, persistent hypotension* and signs of poor peripheral perfusion#. |
| Heart failure | jugular venous distention, new or worsening pulmonary crackles, hypotension, tachycardia, new S3 gallop, new or worsening MR murmur |
| Cardiogenic shock | According to the Society for Cardiovascular Angiography and Interventions  (see Table S5) |

*Hypotension: systolic blood pressure <90 mm Hg; # Signs of poor peripheral perfusion: impaired cognition; cool, clammy, pale, ashen skin; reduced urinary output; peripheral arterial oxygen saturation (SpO_2_) <80% by pulse oximetry; MR = mitral regurgitation

**Table S3. LVEDP-guided hydration protocol**

| **LVEDP (mmHg)** | **Volume expansion rate** |
| --- | --- |
| <13 | 5 mL/kg/h |
| 13-18 | 3 mL/kg/h. |
| >18 | 1.5 mL/kg/h |

LVEDP = left ventricular end diastolic pressure

**Table S4. Algorithm of risk score for contrast-associated acute kidney injury (CA-AKI) proposed by Mehran et al.***

| **Presentation**  **Asymptomatic or stable angina**  **Unstable angina**  **NSTEMI**  **STEMI** | **0**  **2**  **4**  **8** |  | **Score** | **Predicted risk of CA-AKI** |
| --- | --- | --- | --- | --- |
| **eGFR, mL/min/1.73 m^2^**  **≥60**  **30-59**  **<30** | **0**  **1**  **4** |  | 0-2 | 1-2% |
| **Left ventricular ejection fraction <40%** | **2** |  | 3-7 | 2-5% |
| **Age >75 years** | **1** |  | 8-11 | 12-15% |
| **Haemoglobin <11 gr/dL** | **1** |  | ≥12 | 32-35% |
| **Basal glucose ≥150 mg/dL** | **1** |  |  |  |
| **Congestive heart failure on presentation** | **1** |  |  |  |
| **Diabetes mellitus**  **No**  **Non-insulin treated**  **Insulin-treated** | **0**  **1**  **2** |  |  |  |

* According to Mehran et al.([17](#_ENREF_17)) NSTEMI = Non-ST elevation myocardial infarction; STEMI = ST elevation myocardial infarction; eGFR: estimated glomerular filtration rate; Congestive heart failure = class III/IV by New York Heart Association classification and/or history of pulmonary edema.

**Table S5. Algorithm of risk score for contrast-associated acute kidney injury (CA-AKI) proposed by Gurm et al.***

|  | **Risk of CA-AKI** | **Risk of dialysis** |
| --- | --- | --- |
| Low | <1% | <0.10% |
| Intermediate | 1-7% | 0.10-1.5% |
| High | >7% | >1.5% |

The risk of CA-AKI and dialysis can be calculated using a the following website computational tool: <https://bmc2.org/calculators/cin>, according to Gurm et al.([18](#_ENREF_18))

**Table S6. Classification of shock stages***

| **Stage** | **Physical exam/bedside findings** | **Biochemical markers** | **Hemodynamic** |
| --- | --- | --- | --- |
| **A**  **At risk** | Normal JVP  Lung sounds clear  Warm and well perfused  -Strong distal pulses  -Normal mentation | Normal labs  -Normal renal function  -Normal lactic acid | Normotensive (SBP≥100 or normal for pt.)  If hemodynamic done  -cardiac index ≥2.5  -CVP <10  -PA sat ≥ 65% |
| **B**  **Beginning CS** | Elevated JVP  Rales in lung fields  Warm and well perfused  -Strong distal pulses  -Normal mentation | Normal lactate  Minimal renal function impairment  Elevated BNP | SBP<90 or MAP <60 or >30 mmHg drop from baseline  Pulse ≥100  If hemodynamic done  -cardiac index ≥2.2  -PA sat ≥ 65% |
| **C**  **Classic CS** | May include any of:  Looks unwell  Panicked  Ashen, mottled, dusky  Volume overlad  Extensive rales  Killip class 3 or 4  BiPap or mechanical ventilation  Cold, clammy  Acute alteration in mental status  Urine output <30 mL/h | May include any og:  Lactate ≥2  Creatinine doubling OR >50% drop in GFR  Increased LFTs  Elevated BNP | May include Any of:  SBP<90 or MAP <60 or >30 mmHg drop from baseline AND drugs/device used to maintain BP above these targets  Hemodynamics:  -cardiac index <2.2  -PCWP >15  RAP/PCWP ≥0.8  PAPI <1.85  Cardiac power output ≤0.6% |
| **D**  **Deteriorating/doom** | Any of stage C | Any of stage C AND Deteriorating | Any of stage C AND:  Requiring multiple pressors OR addition of mechanical circulatory support devices to maintain perfusion |
| **E**  **Extremis** | Near pulselessness  Cardiac collapse  Mechanical ventilation  Defibrillator used | “Trying to die”  CPR (A-modifier)  pH ≤7.2  Lactate ≥5 | No SBP without resuscitation  PEA or refractory VT/VF  Hypotension despite maximal support |

According to the Society for Cardiovascular Angiography and Interventions (SCAI) classification (23). JVP = jugular vein pressure; <BNP = brain natriuretic peptide; SBP = systolic blood pressure; MAP = mean arterial pressure; CVP = central vein pressure; CPR =cardiopulmonary resuscitation; LFT = liver function tests; PA = pulmonary artery; PCWP = pulmonary capillary wedge pressure; PEA = Pulseless electrical activity; RAP = right arterial pressure; PAPI = pulmonary arterial pressure index; VT = ventricular tachycardia; VF = ventricular fibrillation.

**Table S7. Contrast media thresholds for AKI prevention.**

| **Threshold** | **Reference** |
| --- | --- |
| 3 x GFR | Gurm HS et al. J Am Coll Cardiol 2013 (1) |
| 2.5 x CrCl | Gurm HS, et al. J Invasive Cardiol 2016 (2) |
| V/CrCl >3.7 | Laskey WK et al. (3) |
| 5 mL x body weight /baseline sCr | Freeman RV et al. Am J Cardiol 2002 (4) |
| 140 mL | Briguori C et al. J Am Coll Cardiol 2002 (5) |
| 100 mL | Gurm HS et al. J Am Coll Cardiol 2011 (6) |

GFR = glomerular filtration rate; V = contrast media volume; CrCl = creatinine clearance; sCr = serum creatinine

1. Gurm HS, Seth M, Kooiman J, Share D. A novel tool for reliable and accurate prediction of renal complications in patients undergoing percutaneous coronary intervention. J Am Coll Cardiol 2013;61:2242-8.
2. Gurm HS, Seth M, Mehran R, et al. Impact of Contrast Dose Reduction on Incidence of Acute Kidney Injury (AKI) Among Patients Undergoing PCI: A Modeling Study. J Invasive Cardiol 2016;28:142-6.
3. Laskey WK, Jenkins C, Selzer F, et al. Volume-to-creatinine clearance ratio: a pharmacokinetically based risk factor for prediction of early creatinine increase after percutaneous coronary intervention. J Am Coll Cardiol 2007;50:584-90
4. Freeman RV, O'Donnell M, Share D, et al. Nephropathy requiring dialysis after percutaneous coronary intervention and the critical role of an adjusted contrast dose. Am J Cardiol 2002;90:1068-73
5. Briguori C, Manganelli F, Scarpato P, et al. Acetylcysteine and contrast agent-associated nephrotoxicity. J Am Coll Cardiol 2002;40:298-303.
6. Gurm HS, Dixon SR, Smith DE, et al. Renal function-based contrast dosing to define safe limits of radiographic contrast media in patients undergoing percutaneous coronary interventions. J Am Coll Cardiol 2011;58:907-14

**DyeVert Plus EZ**

The DyeVert™ Plus EZ Contrast Reduction System is intended to reduce the amount of contrast media administered during procedures requiring the injection of contrast media.

The Osprey Medical DyeVert™ Plus EZ Contrast Reduction System is a compatible device to manual contrast injections and provides fluid pathway resistance modulation such that excess contrast volume (i.e., contrast that is not needed for diagnostic or therapeutic purposes) is minimized in the patient’s vasculature and total contrast agent volume reduction occurs; while maintaining adequate image quality.

The DyeVert Plus EZ Contrast Reduction System consists of the Contrast Monitoring Display and the DyeVert Plus EZ Disposable Kit (Smart Syringe and DyeVert Plus EZ Module}. The DyeVert Plus EZ Disposable Kit is intended to be used with the Display to allow monitoring and display of contrast volumes manually injected. Volumes are displayed and compared to physician entered contrast usage thresholds during angiographic procedures.


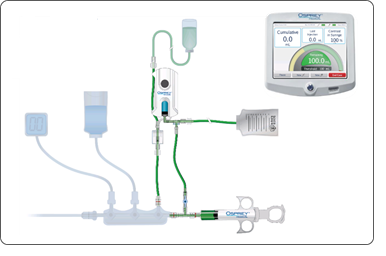

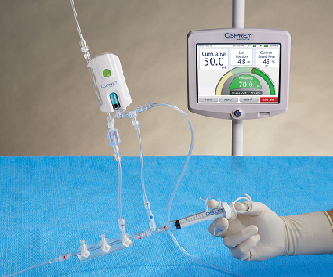


**DyeVert Power XT**

The DyeVert™ Power XT Contrast Reduction System is intended to reduce the amount of contrast media administered to the patient during angiographic procedures using automated injections of contrast media

The Osprey Medical DyeVert™ Power XT Contrast Reduction (DyeVert Power XT) System is compatible with power injectors used in angiographic procedures using contrast media and provides fluid pathway resistance modulation such that excess contrast volume (i.e., contrast for diagnostic or therapeutic purposes that is without clinical benefit) is minimized in the patient’s vasculature and total contrast media dosage reduction occurs; while maintaining adequate image quality.

The DyeVert Power XT is a disposable, single-use sterile device consisting of a diversion line and two catheter size-dependent Diversion Valves. The device is positioned between the power injector’s most proximal connector and the angiographic catheter via the DyeVert Power XT Stopcock. Each of the Diversion Valves responds to the contrast injection pressure and modulates the amount of contrast diverted. The diverted contrast is collected in the Smart Bag.

*Steering Committee*

Carlo Briguori, Giuseppe Massimo Sangiorgi, Gerolama Condorelli, Giovanni Esposito

*Data Monitoring and Safety Committee*

Antonietta Di Iorio (chairperson), Enrica Mariano, Francesca De Micco

*Clinical Event Committee*

Nicola Maurea (chairperson), Fulvio Casale, Stefano Lepore, Giovanni Napolitano

*Clinical Trial and Evaluation Unit Members*

Carlo Briguori (project manager), Carmen D’Amore, Chiara Sordelli, Marco Buonfantino (nephrologist), Giuseppe Signoriello (statistician)
